# Impact of physical activity associated with bariatric surgery on systemic arterial hypertension control: a systematic review

**DOI:** 10.1590/1516-3180.2025.3540.23022026

**Published:** 2026-06-29

**Authors:** Julia Barros Brito, Ana Gabriela Terencio de Sousa, Lélia Lessa Teixeira Pinto, Eric Simas Bomfim, João Henrique Cerqueira Barros, Josias Melo Leite, Luiz Alberto Bastos de Almeida, Lucas Antônio Jesus de Souza, Milton Rocha Moraes, Clarcson Plácido Conceição dos Santos

**Affiliations:** I Undergraduate student. Escola Bahiana de Medicina e Saúde Pública (EBMSP), Salvador (BA), Brazil.; II Undergraduate student. Escola Bahiana de Medicina e Saúde Pública (EBMSP), Salvador (BA), Brazil.; III Assistant teacher, Escola Bahiana de Medicina e Saúde Pública (EBMSP), Salvador (BA), Brazil.; IV Master’s student. Escola Bahiana de Medicina e Saúde Pública (EBMSP), Salvador (BA), Brazil.; V Escola Bahiana de Medicina e Saúde Pública (EBMSP), Salvador (BA), Brazil.; VI Doctorate’s student. Escola Bahiana de Medicina e Saúde Pública (EBMSP), Salvador (BA), Brazil.; VII Professor, Universidade Estadual de Feira de Santana (UEFS), Feira de Santana (BA), Brazil.; VIII Master’s student. Assistant teacher, Escola Bahiana de Medicina e Saúde Pública (EBMSP), Salvador (BA), Brazil.; IX Assistant teacher, Departamento de Educação Física, Centro de Ciências da Saúde, Universidade da Paraíba (UFPB), João Pessoa (PB), Brazil.; X Escola Bahiana de Medicina e Saúde Pública (EBMSP), Salvador (BA), Brazil.

**Keywords:** Exercise, Hypertension, Bariatric surgery, Blood pressure control, Lifestyle intervention, Weight-loss surgery outcomes, Cardiovascular risk reduction

## Abstract

**BACKGROUND:**

Obesity is a highly prevalent condition frequently associated with systemic arterial hypertension (SAH). Bariatric surgery (BS) is an effective strategy for weight loss and has been shown to improve blood pressure (BP) control, whereas physical activity (PA) is recognized as an important adjuvant therapy for treatment of SAH. Nevertheless, evidence regarding the combined impact of BS and PA on BP reduction remains inconsistent.

**OBJECTIVES:**

This review aimed to evaluate whether BS combined with PA contributes to additional BP reduction in individuals with obesity.

**METHODS:**

The review followed PRISMA guidelines and was registered in PROSPERO (CRD42024628299). Eligible studies included randomized controlled trials, cohort and cross-sectional studies involving adults who underwent BS, with or without PA. Searches were performed in CENTRAL, PubMed, LILACS, and BVS. Methodological quality was assessed using the RoB2 and JBI tools, and the certainty of evidence was graded according to GRADE.

**RESULTS:**

Of the 406 records screened, nine studies were included (n = 504 participants). BS alone was associated with significant reductions in BP. When PA was combined with BS, additional reductions were reported; however, the findings were heterogeneous and supported by low to very low certainty of evidence. The follow-up duration across studies ranged from 4 months to 5 years.

**CONCLUSION:**

The combination of BS and PA provides modest but clinically relevant benefits in BP reduction. However, the limited number of studies and short follow-up periods preclude definitive conclusions. High-quality, long-term randomized clinical trials are warranted to clarify the role of PA in optimizing BP control after BS.

**CLINICAL TRIAL OR SYSTEMATIC REVIEW REGISTRATION:**

The review followed PRISMA guidelines and was registered in PROSPERO (CRD42024628299).

## INTRODUCTION

 Obesity represents one of the most pressing public health challenges worldwide. According to the Pan American Health Organization, approximately one in every eight individuals is affected, corresponding to over 1 billion people currently living with the condition.^1^

 Beyond its high prevalence, obesity substantially increases the risk of systemic arterial hypertension (SAH) and contributes to the overall cardiovascular burden. It is associated with dyslipidemia, atrial fibrillation, heart failure, stroke, insulin resistance, and other cardiometabolic alterations, all of which are strongly linked to higher all-cause mortality.^1^

 Elevated blood pressure (BP) in overweight individuals or individuals with obesity results from multiple mechanisms. The key factors include renal compression by perirenal/intrarenal fat, impaired sodium excretion; insulin resistance, hyperinsulinemia, and obstructive sleep apnea.^2^ Additional contributors include heightened sympathetic and renin-angiotensin-aldosterone system activity, altered baro- and chemoreceptor function, chronic adipokine-mediated inflammation, and hyperuricemia linked to high-fructose intake, which promotes oxidative stress and endothelial dysfunction.^3 -5^ These mechanisms highlight the role of visceral obesity in SAH, indicating that excess weight plays a central role. 

 In this context, bariatric surgery (BS) emerges as an alternative to address these frequently coexisting comorbidities, obesity and SAH. Beyond weight loss, patients undergoing this procedure experience a significant decrease in systolic blood pressure (SBP) and diastolic blood pressure (DBP).^6^ Furthermore, studies have shown that BS also promotes a significant decrease in mean arterial pressure (MAP). 

 Several studies have compared BS with drug therapy in patients with hypertension and have showed significant benefits: BS leads to a greater reduction in the use of antihypertensive medication in the medium- and long-term compared to drug therapy alone.^7,8^

 In contrast, physical activity (PA) is a widely recognized intervention for cardiovascular and cardiorespiratory benefits,^9^ and is a crucial option for patients with obesity and SAH. However, candidates for BS are less physically active and engage in fewer minutes of PA per day than the general population. Furthermore, they show lower adherence rates to PA,^10^ which limits the benefits of this measure. 

 Studies have indicated that participation in PA after BS is associated with improvements in muscle strength and cardiorespiratory and physical functions.^11,12^ Published reviews have suggested that PA as an adjunct to BS is associated with improved weight loss and quality of life,^13^ as well as improved BP levels.^14^

## OBJECTIVE

 Although several studies have addressed the benefits of PA combined with BS,^15-18^ evidence remains inconsistent. Furthermore, there is a scarcity of high-quality studies that specifically evaluate whether PA enhances BP reduction in this context. Given this gap in knowledge, the objective of this study was to assess, through a systematic review, whether PA combined with BS contributes to BP reduction in people with obesity. 

## METHODS

### Protocol and registration

 This systematic review was conducted in accordance with the Cochrane Handbook for Systematic Reviews of Interventions and the Preferred Reporting Items for Systematic Reviews and Meta-Analysis (PRISMA) guidelines. The protocol was registered in the International Prospective Register of Systematic Reviews (PROSPERO: CRD42024628299). 

### Inclusion criteria

 Studies were selected based on the following inclusion criteria: (1) randomized controlled trials, cross-sectional studies, and cohort studies including adult patients (≥ 18 years) who underwent BS, with no restrictions regarding drug therapy for comorbidities; and (2) no restrictions on the year of publication or language. 

### Exclusion criteria

 The exclusion criteria were as follows: (1) systematic reviews or studies using secondary data; and (2) studies involving individuals with neurological and/or psychiatric impairments. 

### Search and data extraction strategy

 The final search for studies evaluating the effects of BS, whether associated with PA or not, was conducted on December 18, 2024, in the following databases: Cochrane Central Register of Controlled Trials (CENTRAL), PubMed, LILACS, and the Virtual Health Library (BVS). The PICOS framework guided the research question: In patients who underwent BS, does PA help reduce BP levels? The inclusion criteria were as follows: (population: patients who underwent BS; intervention: practice of PA; comparison: patients who underwent BS without PA; outcome: reduction of SBP and DBP). The keywords and Medical Subject Headings (MeSH) terms were “physical activity,” “exercise,” “blood pressure,” “hypertension,” and “bariatric surgery.” Two authors, independently and blindly, assessed the studies in two stages. In the first stage, titles, abstracts, and duplicate studies were screened for eligibility using the Rayyan QCRI application (Qatar Computing Research Institute, Doha). Subsequently, the full texts were evaluated for eligibility. Disagreements were resolved by consensus or by a third reviewer. Data extracted included the authors, country, year, sample size, study design, objectives, intervention type, assessed parameters, treatment duration, and results. 

### Risk of bias assessment

 Methodological quality was assessed using the Cochrane Risk-ofBias 2 (Rob 2) tool, developed for randomized clinical trials, and the Joanna Briggs Institute (JBI) Risk-of-Bias tool, developed to analyze the risk of bias in cross-sectional and cohort studies. Bias domains included randomization, deviations from intended interventions, outcome measurement, missing outcome data, and selection of reported results, which were classified as low, with some concerns, or high. Disagreements were resolved by discussion. 

 The certainty of evidence regarding the impact of PA on BP reduction after BS was assessed using the GRADEpro Guideline Development Tool (software), considering risk of bias, inconsistency, indirectness, imprecision, and publication bias. Evidence quality was classified as high, moderate, low, or very low. 

## RESULTS

### Characteristics of the included studies

 Participant and outcome assessor blinding were not feasible for the proposed intervention (PA), which may have compromised the risk of bias assessment, as performance bias was considered high. Consequently, the certainty of the evidence was rated as very low to low according to the GRADE approach (Grading of Recommendations Assessment, Development, and Evaluation) (**Table 1**). 

**Table 1:** Analysis of risk of bias using GRADE

**Certainty assessment**							**N^º^ of patients**			**Effect**		**Certainty**	**Importance**
**N^º^ of studies**	**Study design**	**Risk of bias**	**Inconsistency**	**Indirect evidence**	**Imprecision**	**Other considerations**	**bariatric surgery and physical exercise**	**Patiens without physical exercise**		**Relative (95% CI)**	**Absolute (95% CI)**		
**Cohort 4**		Observational study	Not serious	Not serious	Serious		Serious	None	0/250	0/250	Not estimable	Very low	CRITICAL
**Clinical trials 3**		Randomized clinical trials	Not serious	Not serious	Serious		Serious	None	0/70	0/70	Not estimable	Low	CRITICAL
**Cross-sectional 2**		Observational studies	Not serious	Not serious	Not serious		Serious	None	0/32	39/0	Not estimable	Very low	CRITICAL

**Figure 1:**
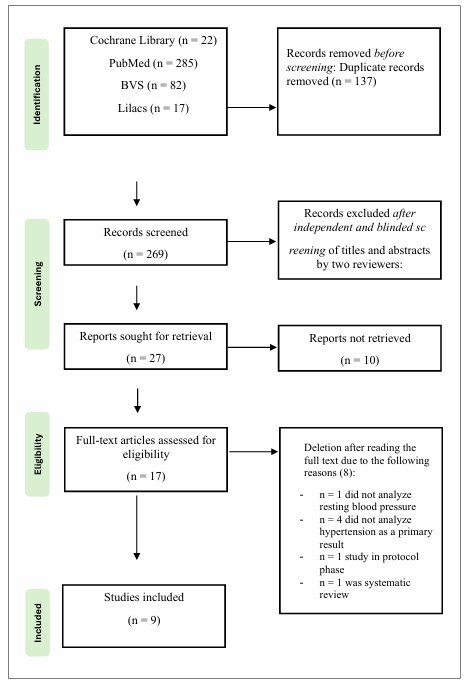
Primary flowchart of the study selection process for inclusion in the systematic review.

 A total of 406 studies were identified through the search strategy (**Figure 1**). After screening the titles and abstracts, 269 articles were retained, of which seventeen were considered potentially eligible for full-text review; eight were subsequently excluded for not meeting the eligibility criteria. Ultimately, nine studies were included in this systematic review (**Figure 1** and **Table 2**). 

**Table 2:** Characteristics of the studies included in the systematic review

**Author/year**	**Study design**	**Sample (n)**	**Age range/average age**	**Intervention/physical activity**	**Follow-up period**
Keleidari et al., 2016	Cross-sectional	35	31,45 ± 8, 84 years	Not specified	6 months
Serés et al., 2006	Prospective	31	38 ± 8 years	Not specified	1 year
Moriconi et al., 2024	Observational, 5 years	148	54 ± 9 years (DM2); 45 ± 10 years (without DM2)	≥ 150 min per week of moderate aerobic activity + 2–3 sessions of muscle strengthtening	5 years
Mundbjerg et al., 2018	Randomized controlled clinical trial	60	42,3 ± 9, 1 years	Supervised training twice per week, 40 min (15 min cycling, 10 min Upper limb strength, 15 min free exercise)	2 years
Pereira et al., 2019	Retrospective cross-sectional	78	37–45 years	Not specified	1 year
Jankiewicz-Wika et al., 2011	Prospective cross-sectional	28	43,7 ± 10 years (20–59 years)	Not specified	2 years
Belzile et al., 2023	Randomized clinical trial	59	42 years	Supervised training 3 times per week for 12 weeks, 60 min (35 min aerobic + 25 min strength exercise)	1 year
Valezi et al., 2011	Observatoinal, longitudinal, analytical, prospective	43	35,9 ± 12, 2 years	Not specified	1 year
Castello et al., 2010	Prospective randomized controlled	21	20–45 years	Aerobic treadmill training, 36 sessions for 12 weeks (60 min on alternate days)	4 months

 The included studies, published between 2006 and 2024, were conducted in Brazil (n = 3), Iran (n = 1), Italy (n = 1), Spain (n = 1), Poland (n = 1), Canada (n = 1), and Denmark (n = 1), encompassing a total of 504 participants, of whom 368 were women (73%). Four studies compared the combined effects of BS and PA. 

 The studies were divided into subgroups: four evaluated BS associated with or without supervised PA, and five assessed body changes after BS and their effects on hypertension, specifically SBP and DBP (**Table 3**). 

**Table 3:** Variations of blood pressure after bariatric surgery

**Study**	**Time**	**Group**	**Pre SBP**	**Post SBP**	**Δ SBP**	**Pre DBP**	**Post DBP**	**Δ DBP**	**Observations**
Keleidari et al., 2016	6 months	–	124.4 ± 7.8	116.6 ± 5.6	−7.8	79.8 ± 5	76.8 ± 5.3	−3	Significant reduction
Serés et al., 2006	12 months	–	135 ± 18	127 ± 17	−8	87 ± 11	77 ± 12	−10	Reduction after 1 year
Moriconi et al., 2024	5 years	PA	137 ± 6	122 ± 9	−15	84 ± 6	75 ± 7	−9	Sustained reductoin
Mundbjerg et al., 2018	12 months	Intervention	–	–	–	–	−4.8*	–	Reduction in DBP
Pereira et al., 2019	Up to 6 months	BS2 to BS+6	–	–	–	–	–	–	Only reported body weight
Jankiewicz-Wika et al., 2011	24 months	–	↓	↓	↓	↓	↓	↓	BP values were not reported
Belzile et al., 2023	3–12 months	–	–	–	–	–	–	–	Clinical remission of hypertension
Valezi et al., 2011	12 months	–	–	–	−7,7%	–	–	NS	Reduction only in SBP; DBP with no changes
Castello et al., 2010	12 weeks	Intervention	150 ± 7.1	146.6 ± 4	−3.4	88.8 ± 2.4	85 ± 3	−3.8	Effect only on intervention

Δ SBP/DBP: difference between the pre- and postoperative values (absolute value);

↓ indicates a significant reduction without exact numerical data;

* Mundbjerg et al., 2018: only DBP showed a significant difference (−4.8 mmHg in the intervention group);

SBP remained stable; Pereira et al., 2019: focused on weight loss and did not report BP; Belzile et al., 2023: clinical improvement in hypertension, but without numerical data.

### Body and metabolic changes after bariatric surgery

 In the study by Keleidari et al.,^19^ 35 individuals with obesity underwent BS and were followed for 6 months. Significant reductions in SBP and DBP were observed (SBP: from 124.4 ± 7.8 mmHg to 116.6 ± 5.6 mmHg; DBP: from 79.8 ± 5 mmHg to 76.8 ± 5.3 mmHg) (p < 0.05), with a mean decrease of 4.6 mmHg in MAP. 

 In the study by Serés et al.,^20^ 31 patients with morbid obesity were followed for one year after undergoing BS. SBP decreased from 135 ± 18 to 127 ± 17 mmHg (p = 0.07), whereas DBP showed a more pronounced reduction, from 87 ± 11 to 77 ± 12 mmHg (p < 0.001). During maximal effort, SBP remained unchanged, whereas DBP decreased significantly. The MAP decreased both at rest and during maximum effort (103–93.7 mmHg and 127.3–123.7 mmHg). 

 Pereira et al.,^11,21^ followed 78 patients undergoing BS, grouped according to the postoperative time (BS2:1–2 years; BS4:2–4 years; BS6:4–6 years; BS+6:6–10 years) and assessed comorbidities using the ACRO score, an adapted cardiorespiratory fitness score for obesity, developed to evaluate functional capacity before and after BS. All groups showed improvement in obesity-related conditions, particularly in hypertension. The proportion of participants with controlled hypertension (ACRO ≤ 2) increased from 8.3% to 66.6% in BS2, from 64.2% to 92.8% in BS4, from 40.9% to 81.8% in BS6, and from 23.3% to 86.6% in BS+6, indicating sustained improvement in BP control over time. 

 Jankiewicz-Wilka et al.,^22^ evaluated 28 patients with morbid obesity and metabolic syndrome after BS with a follow-up of up to 48 months. After 24 months, a significant reduction in body mass index (BMI), waist circumference, and BP was observed, with a gradual decrease over time (p < 0.05 for SBP and p < 0.01 for DBP), although absolute BP values were not reported. 

 In contrast, Valezi et al.,^23^ analyzed 43 patients with class III obesity before and 12 months after Roux-en-Y gastric bypass (RYGB). Significant reductions were observed in body weight (from 116.5 ± 21.5 kg to 80 ± 15.9 kg), BMI (from 41.8 ± 4.4 kg/m^2^ to 28.4 ± 3.8 kg/m^2^), and SBP from 130 mmHg to 120 mmHg (p < 0.001), while DBP remained unchanged (p > 0.05). The MAP decreased from 96.7 mmHg to 93.3 mmHg. The follow-up periods in surgical-only studies ranged from 2 weeks to 24 months. 

### Body and metabolic changes in bariatric surgery associated with physical activity

 Moriconi et al.,^24^ evaluated the impact of PA on BP control in individuals with and without type 2 diabetes mellitus (T2DM). The intervention included ≥ 150 min/week of moderate-intensity aerobic activity plus strength training. After five years, active participants with T2DM showed lower SAH prevalence (33% versus 62%; p = 0.0043), reductions in SBP, DBP, and MAP (approximately 102–92 mmHg), and decreased antihypertensive medication use (73% to 33% in the active group versus 78.7% to 62.3% in the control group). Among participants without T2DM, SAH prevalence was also lower in active individuals (6.9% versus 28%), with levels sustained throughout the follow-up. Overall, PA was consistently associated with lower BP values, independent of medication use (p < 0.001). 

 Mundbjerg et al.,^25^ evaluated a supervised PA program in 44 participants after BS and divided them into intervention and control groups. The intervention combined aerobic and resistance training for 26 weeks. After 24 months, no significant difference in SBP was observed; however, DBP was significantly lower in the intervention group (difference of 4.8 mmHg; p = 0.034), indicating a sustained benefit of PA. The MAP decreased from approximately 103 mmHg to 94 mmHg, reflecting clinically relevant hemodynamic improvement. 

 In a randomized study including 59 patients with severe obesity who underwent BS, the participants were assigned to a control or a 12-week supervised exercise program. Baseline SAH prevalence was similar between both groups (36.8% in the control group and 42.5% in the intervention group; p = 0.62). Most comorbidity resolutions, including hypertension, occurred within the first three months post-surgery, with no significant differences between groups thereafter (p > 0.05).^26^

 Castello et al.,^27^ randomized 21 women to control or supervised 12 weeks of aerobic training after BS. SBP was 150 ± 7.1 mmHg in the control group and 146.6 ± 4 mmHg after intervention, while DBP was 88.8 ± 2.4 mmHg and 85 ± 3 mmHg, respectively. Both groups showed a significant reduction in DBP. The MAP was 109 mmHg in the control group and 105.5 mmHg in the intervention group. 

**Figure 2:**
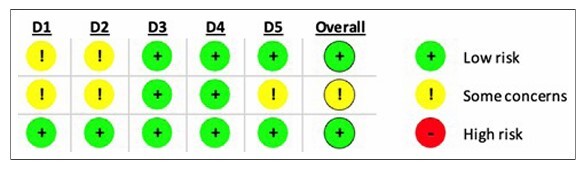
Analysis of risk of bias using the platform RoB-2.

 Regarding methodological quality, three randomized trials were assessed using the RoB2 tool (**Figure 2**) developed by Cochrane for randomized clinical trials, while the remaining six studies were assessed using the JBI tool, which provides standardized instruments for evaluating different study designs in systematic reviews. Regarding randomized clinical trials, according to the RoB2 tool, two studies had a low risk of bias, while one had a moderate risk. Analysis using the JBI platform revealed that one study had a reliability rating of 90.9 %, while four studies had 72.7% and one had a 63.6%. 

## DISCUSSION

 To our knowledge, this is the first review to provide evidence synthesis examining the effectiveness of PA combined with BS in reducing SAH, thereby addressing an important gap in the literature. 

 This review included three randomized clinical trials,^25-27^ two cross-sectional studies,^19,21^ and four cohort studies.^20,22 -24^ Among these, three trials^25-27^ and one cohort study^24^ specifically evaluated the effect of PA on BP, whereas the remaining studies analyzed the effects of BS alone. 

 Although BP changes before and after BS were assessed, few studies reported post-intervention values in sufficient details, thus preventing a meta-analysis as outlined in the protocol. 

 Overall, the findings regarding the impact of PA on BP reduction after BS were inconsistent. Belzile et al.,^26^ reported that comorbidity resolution occurred mainly shortly after surgery, with no additional benefit from PA (p > 0.05). Conversely, Mundbjerg et al.^25^ identified a significant reduction in DBP in the PA group after 24 months, with a difference of 4.8 mmHg compared to the control group (p = 0.034). 

 Castello et al.,^27^ despite analyzing the results only four months after BS,^28^ observed a significant reduction in SBP in both groups. However, a significant reduction in DBP was recorded only in the PA group. Moriconi et al.^24^ consistently reported lower BP values among patients who engaged in PA in all analyzed periods (p < 0.001). 

### Quality of evidence

 The risk of bias assessment revealed recurrent limitations, including inadequate randomization and the impossibility of blinding owing to the nature of PA interventions. Insufficient methodological details made it difficult to assess bias, leading to a downgrading of the evidence using the GRADE tool. Additionally, small sample sizes (22–148 participants) and the limited number of studies specifically evaluating PA^24 -27^ further reduced the robustness of the findings. 

### Agreements and disagreements with other studies or reviews

 There was no consistency among the studies regarding whether PA helped reduce BP when combined with BS. 

 Few studies have directly evaluated the impact of PA on BP after BS, which limited the available data and required cautious interpretation. 

 BS induces important physiological changes, with BP reduction frequently observed. Serés et al.^20^ and Huang et al.^29^ reported greater reductions in DBP than in SBP, the latter often without statistical significance. 

 This pattern may reflect the higher sensitivity of DBP to early metabolic and vascular changes. According to the American Heart Association,^28^ DBP decreases earlier due to reduced peripheral resistance and inflammation, whereas SBP depends on slower changes in arterial compliance and stiffness.^28^

 Similarly, Van Brussel et al.^30^ highlighted that DBP responds more rapidly to hemodynamic variations, whereas SBP reflects later structural adaptations. 

 Conversely, Valezi et al.^23^ observed a stable DBP with changes in SBP only. The mechanisms explaining the isolated decrease in SBP include increased arterial compliance and reduced systolic pressure peaks, without vasodilation of the microcirculation.^31^ Further analysis of this study outcome suggests that one possible explanation is that baseline DBP was already within normal limits, which may have limited the observation of a significant decrease. 

 Three studies demonstrated reductions in both SBP and DBP.^19,21 ,22^ The long follow-up period in the Jankiewicz-Wilka et al. study^22^ allowed the observation of sustained metabolic and hemodynamic effects, while higher baseline BP levels across these studies may have favored reductions in both parameters. 

 Regarding PA, of the four studies evaluated, one showed no post-intervention changes;^26^ one showed changes in both parameters;^24^ and two showed changes in DBP only.^25,27^

 Evidence suggests that most BP benefits occur after BS in the short term, with a limited additional impact from supervised PA. Belzile et al.^26^ observed the resolution of hypertension mainly in the immediate postoperative period, without further changes after PA introduction. Similarly, Chen Hu et al.^32^ attributed BP and BMI reductions primarily to BS rather than PA. 

 Consistent with these findings, Carretero-Ruiz et al.,^33^ found no significant reductions in BP associated with PA in bariatric patients. Despite the overall beneficial effects of BS, the association between PA and BP control in the immediate postoperative period has not yielded consistent results. 

 Castello et al.^27^ initiated PA interventions early, immediately after BS, which may have influenced the results; in contrast, Ren et al.^14^ reported significant BP reductions when PA was initiated one year after surgery. 

 In other studies, BP reduction has been observed in both SBP and DBP values. Moriconi et al.^24^ reported significant reductions in SAH in both patients with and without T2DM, whereas Ren et al.,^14^ showed greater weight loss and lower BP among physically active patients. 

 In contrast, other studies have reported significant changes only in DBP following supervised PA. Castello et al.^27^ observed reductions mainly in DBP compared with SBP. Mundbjerg et al.^25^ found that, 24 months after surgery and with regular exercise practice, changes occurred primarily in DBP. This may be explained by the fact that Castello’s intervention was short-term and of moderate intensity, which tends to affect peripheral resistance (related to DBP) more than central arterial compliance (related to SBP). 

 The long-term BP effects after BS vary with follow-up duration. Climent et al.,^34^ investigated the impact of BS on BP across different follow-up periods. In the short term (less than three years), BS showed a strong association with SAH remission. However, the results in the medium term (3–5 years) and long term (more than five years) were less consistent and demonstrated modest effect sizes. 

 One included study with a 36-month follow-up showed that 68.1% of hypertensive patients achieved BP remission in the first postoperative year; however, 21.9% experienced recurrence by three years. Similar relapse patterns were reported by Bäckdahl et al. ,^35^ in the GATEWAY trial, a randomized clinical trial that evaluated the effect of BS in patients with pharmacologically controlled SAH, in which 80% of patients undergoing BS reduced antihypertensive use at 12 months, compared with 13% in the control group. BP reduction occurred early and stabilized around the sixth month, while a five-year follow-up revealed hypertension recurrence in approximately 20% of patients. 

 Among the studies included, Castello et al.^27^ had the shortest follow-up, with only four months after BS, whereas JankiewiczWilka et al.^22^ had the longest follow-up period (two years). Thus, none assessed medium- or long-term effects, limiting conclusions regarding durability and potentially underestimating recurrence rates. 

## CONCLUSION

 Current evidence indicates that BS combined with PA may promote modest yet clinically relevant reduction in BP. However, the limited number of studies, methodological heterogeneity, and short follow-up periods preclude definitive conclusions, highlighting the need for larger, high-quality studies. 

## Data Availability

Data supporting the findings of this study are available upon request from the corresponding author, Clarcson Plácido Conceição dos Santos.
